# Risk factors associated with mortality among patients who had candidemia in a university hospital

**DOI:** 10.1590/0037-8682-0206-2019

**Published:** 2020-06-22

**Authors:** Priscila Guerino Vilela Alves, Sávia Gonçalves Oliveira Melo, Meliza Arantes de Souza Bessa, Murilo de Oliveira Brito, Ralciane de Paula Menezes, Lúcio Borges de Araújo, Mário Paulo Amante Penatti, Reginaldo dos Santos Pedroso, Denise Von Dolinger de Brito Röder

**Affiliations:** 1 Universidade Federal de Uberlândia, Faculdade de Medicina, Uberlândia, MG, Brasil.; 2 Universidade Federal de Uberlândia, Instituto de Biologia, Uberlândia, MG, Brasil.; 3 Universidade Federal de Uberlândia, Instituto de Geografia, Uberlândia, MG, Brasil.; 4 Universidade Federal de Uberlândia, Escola Técnica de Saúde, Uberlândia, MG, Brasil.; 5 Universidade Federal de Uberlândia, Faculdade de Matemática, Uberlândia, MG, Brasil.; 6 Universidade Federal de Uberlândia, Instituto de Ciências Biomédicas, Uberlândia, MG, Brasil.

**Keywords:** Candidemia, Risk factors, Mortality

## Abstract

**INTRODUCTION:**

Bloodstream infection due to *Candida* spp. is a primary cause of morbidity and mortality in tertiary hospitals.

**METHODS:**

In this retrospective study, we included patients with a positive blood culture for *Candida* spp. after 48 h of hospitalization.

**RESULTS:**

A total of 335 patients who had candidemia were included in this study. Risk factors associated with mortality were hospitalization in internal medicine units and surgical clinics, age >60 years, mechanical ventilation, orotracheal intubation, hemodialysis, corticosteroids use, and *C. parapsilosis* infection.

**CONCLUSIONS:**

This study highlights the importance of health care related to invasive procedures and actions to improve patient immunity.

Bloodstream infection (BSI) is one of the main causes of morbidity and mortality in tertiary hospitals, and 7.9% to 9.0% of infections are caused by *Candida* spp. Approximately 50% of candidemia cases are caused by *C. albicans*, followed by *C. glabrata* complex, *C. parapsilosis* sensu lato, and *C. tropicalis* ^1^ ^-^ ^3^. 

Episodes of candidemia have occurred mainly among patients who have been hospitalized for long periods of time and have been exposed to antimicrobials drugs, immunosuppressive therapy, parenteral nutrition, and invasive medical procedures. Typically, candidemia has a difficult diagnosis and treatment, a high mortality rate (40% to 60%), and incurs high hospital costs^4^. The incidence rate of candidemia in Brazil ranges from 0.91 to 2.49 per 1000 admissions^5^. This study aimed to evaluate the risk factors associated with mortality in patients who had BSI caused by *Candida* spp. in a Brazilian tertiary care hospital.

A retrospective study was carried out at the Hospital of Clinics of the Federal University of Uberlândia, a tertiary care university hospital with 520 beds located in Minas Gerais in southeastern Brazil. Patients with positive blood cultures for *Candida* spp., obtained after 48 h of hospitalization, between 2009 and 2016 were included in the study. These patients were selected from the database of the Clinical Analysis Laboratory of the hospital.

Data were collected from medical records and included age, sex, hospital sector, comorbidities, invasive procedures, antifungal therapy use, corticosteroids use, prior antimicrobial therapy, length of hospital stay before blood culture positivity, crude mortality rate, and *Candida* species. The incidence rate of candidemia per 1000 admissions was calculated through the following equation: total number of patients that had candidemia/total number of hospitalized patients × 1000. The crude mortality rate was calculated through the following equation: total number of deaths in patients that had candidemia/total number of hospitalized patients during the study period x 1000.

Blood samples were processed in the Microbiology Unit of the Clinical Analysis Laboratory using the BacT/ALERT® 3D system (Biomérieux, France) and identified by traditional methods (chromogenic medium, micromorphological analysis, and staining of Gram); all species were confirmed using the VITEK® 2 system (BD Diagnostic Systems, Franklin Lakes, NJ, USA). 

Qualitative variables were expressed as frequencies and percentages, and quantitative variables were expressed as mean and standard deviation. For univariate and multivariate analyses, logistic regression was used, and a *P*-value ≤0.05 was considered statistically significant. All analyses were performed using SPSS software for Windows (version 20.0; IBM Corp., Armonk, NY, USA).

This study included 335 patients who had candidemia between 2009 and 2016, ranging in age from 1 day to 96 years. The incidence of candidemia was 1.36 infections per 1000 admissions, and the crude mortality rate was 54.6%.

Clinical, demographic, and outcome characteristics of the patients are presented in Table 1. The mortality rate was higher in patients aged over 60 years (*P* < 0.01), those who underwent hemodialysis (*P* < 0.01), and those who required mechanical ventilation (*P* < 0.01) or orotracheal intubation (*P* < 0.01) or had *C. parapsilosis* infection (*P* = 0.03) during hospitalization. Corticosteroids use (P < 0.01), hemodialysis (P < 0.01), orotracheal intubation (*P* < 0.01), mechanical ventilation (*P* < 0.01), and (6)*C. parapsilosis* infection (*P* = 0.01) were independent risk factors for mortality. 

The risk factors related to death and hospitalization are shown in Table 2. Patients hospitalized in the internal medicine unit (6)(*P* = 0.03) and surgical clinic (*P* < 0.01) had a higher incidence of mortality. The majority of patients (65.1%, 218/335) required treatment in the intensive care unit (ICU) at some point during hospitalization. Furthermore, the following factors were found to be protective in relation to mortality, with an odds ratio (OR) less than 1.00: hospitalization in the emergency unit or pediatric ICU, use of parenteral nutrition, use of fluconazole or amphotericin B and the duration of their use, and total hospitalization time.

**TABLE 1:** Analysis of clinical and demographic characteristics of patients with candidemia in relation to mortality in a university hospital (2009-2016).

Characteristics	Survived		Death		Univariate		95% Confidence		Multivariate		95% Confidence	
	(n=152)		(n=183)		analysis		interval		analysis		interval	
	N	%	N	%	*P*-value*	OR	Lower	Upper	*P*-value*	OR	Lower	Upper
Age												
0 - 30 days^a^	15	9.9	9	4.9	-	-	-	-	-	-	-	-
31 days- 1 years old	15	9.9	12	6.6	0.61	1.33	0.43	4.10	-	-	-	-
>2 -11 years old	16	10.5	4	2.2	0.21	0.41	0.11	1.64	-	-	-	-
>12-24 years old	12	7.9	0	0	0.99	1.00	-	-	-	-	-	-
>25-39 years old	18	11.8	17	9.3	0.40	1.57	0.55	4.54	-	-	-	-
40-59 years old	49	32.2	52	28.4	0.22	1.76	0.71	4.41	-	-	-	-
>60 years old	27	17.8	89	48.6	<0.01*	5.49	2.16	13.95	-	-	-	-
**Males**	92	50.3	91	59.9	0.07	0.68	0.44	1.04	-	-	-	-
**Surgery of the gastrointestinal tract**	32	21.6	56	30.8	0.06	1.61	0.97	2.66	-	-	-	-
**Comorbidity**												
Renal transplantation	3	2.0	1	0.5	0.26	0.27	0.02	2.65	-	-	-	-
HIV	4	2.8	6	3.3	0.76	1.21	0.33	4.39	-	-	-	-
Neoplasia	26	17.9	44	24.4	0.15	1.48	0.85	2.55	-	-	-	-
Cardiopathy	9	6.2	19	10.6	0.16	1.78	0.78	4.07	-	-	-	-
Diabetes mellitus	16	11.0	24	13.3	0.53	1.24	0.63	2.43	-	-	-	-
Hypertension	30	20.7	48	26.7	0.21	1.39	0.82	2.34	-	-	-	-
Invasive procedures												
Mechanical ventilation	70	47.6	121	66.5	<0.01*	2.18	1.39	3.41	<0.01*	2.18	1.24	3.72
Tracheostomy	31	21.2	38	20.9	0.93	0.97	0.57	1.66	-	-	-	-
CVC	137	93.2	163	89.6	0.25	0.62	0.28	1.39	-	-	-	-
Nasoenteral catheter	85	57.8	108	59.3	0.78	1.06	0.68	1.65	-	-	-	-
Hemodialysis	18	12.3	85	46.7	<0.01*	6.23	3.51	11.05	<0.01*	5.10	2.68	9.73
Parenteral nutrition	71	48.3	63	34.6	0.01*	0.56	0.36	0.88	-	-		
Colostomy bag	7	4.8	14	7.7	0.28	1.66	0.65	4.24	-	-		
Orotracheal intubation	7	4.8	32	17.6	<0.01*	4.26	1.82	9.97	<0.01*	4.93	1.74	14.00
**Antifungal agents**												
Fluconazole	134	89.3	152	83.1	0.10	0.58	0.30	1.11	-	-	-	-
Time of use (days)^b^	11.6±11.2		15.9±17.5		0.01*	0.97	0.95	0.99	0.04*	0.97	0.95	0.99
Amphotericin B	31	20.7	18	9.8	<0.01*	0.41	0.22	0.78	-	-	-	-
Time of use (days)^b^	1.2±5.1		3.3±8.0		<0.01*	0.95	0.91	0.98	-	-	-	-
Micafungin	23	15.3	30	16.4	0.79	1.08	0.59	1.95	-	-	-	-
Time of use (days)^b^	2.1±6.2		2.2±6.0		0.84	0.99	0.96	1.03	-	-	-	-
Use of corticosteroids	70	50.7	126	72.0	0.00*	2.49	1.56	3.99	0.01*	2.23	1.26	3.90
**Species**												
*C. albicans*	70	46.1	84	45.9	0.97	0.99	0.64	1.53	-	-	-	-
*C. tropicalis*	34	22.4	41	22.7	0.99	1.00	0.59	1.68	-	-	-	-
*C. parapsilosis* sensu lato	19	12.5	39	21.3	0.03*	1.90	1.04	3.44	0.01*	2.51	1.17	5.40
*C. krusei*	7	4.6	7	3.8	0.73	0.83	0.28	2.41	-	-	-	-
*C. glabrata* complex	14	9.2	16	8.7	0.88	0.94	0.44	2.00	-	-	-	-
Others**	11	7.2	13	7.1	0.96	0.98	0.42	2.25	-	-	-	-

a reference class for the age groups; ^b^ mean ± standard deviation; *P ≤ 0.05 considered significant; **C. lusitaniae, *C. famata*, *C. guilliermondii*, *Candida* spp., and *C. utilis*. **OR:** odds ratio; **ICU:** intensive care unit; **NICU:** neonatal intensive care unit; **PICU:** pediatric intensive care unit; **AICU:** adult intensive care unit; **CIUC:** coronary intensive care unit; **CVC:** Central Venous Catheters; **HIV:** Human immunodeficiency virus. - data were not shown because they were not significant for a *P* ≤ 0.05.

**TABLE 2:** Analysis of the hospitalization of patients with candidemia in relation to mortality in a university hospital (2009-2016).

Characteristics	Survived		Death		Univariate		95% Confidence		Multivariate		95% Confidence	
	(n=152)		(n=183)		analysis		interval		analysis		interval	
	n	%	N	%	*P*-value*	OR	Lower	Upper	*P*-value*	OR	Lower	Upper
ICU hospitalization	99	65.1	119	65.0	0.98	0.99	0.63	1.56	-	-	-	-
NICU	17	11.2	16	8.7	0.45	0.76	0.37	1.56	-	-	-	-
PICU	17	11.2	7	3.8	0.01*	0.31	0.12	0.78	-	-	-	-
AICU	65	42.8	92	50.3	0.17	1.35	0.87	2.08	-	-	-	-
CIUC	1	0.7	2	1.1	0.67	1.66	0.14	18.58	-	-	-	-
ICU hospitalization time (days)^a^	19.9±30.0		20.0±24.9		0.97	1.00	0.992	1.008	-	-	-	-
Surgical clinic	31	20.4	60	33.0	<0.01*	1.94	1.17	3.20	-	-	-	-
Internal medicine	16	10.5	34	18.6	0.03*	1.97	1.04	3.72	-	-	-	-
Emergency unit	50	32.9	29	16.0	<0.01*	0.39	0.23	0.66	<0.01	0.33	0.19	0.56
Pediatric	18	11.8	4	2.2	<0.01*	0.17	0.06	0.51	<0.01*	0.14	0.05	0.42
Oncology	7	4.6	9	5.0	0.87	1.08	0.39	2.98	-	-	-	-
Others	13	8.6	17	9.3	0.78	1.11	0.52	2.36	-	-	-	-
Hospitalization time before positive culture (days)^a^	32.9±44.3		36.6±51.8		0.45	0.999	0.99	1.003	-	-	-	-
Total hospitalization time (days)^a^	56.0±76.6		74.9±51.4		0.01*	0.999	0.99	0.999	0.04*	0.996	0.992	0.999

a mean ± standard deviation; *P ≤ 0.05 considered significant; **OR:** odds ratio; **ICU:** intensive care unit; **NICU:** neonatal intensive care unit; **PICU:** pediatric intensive care unit; **AICU:** adult intensive care unit; **CIUC:** coronary intensive care unit. - data were not shown because they were not significant for a *P* ≤ 0.05.

Antimicrobial therapy use before diagnosis was documented in 97.6% of patients. The most commonly used antifungal treatment was fluconazole (85.4%, 286/335 patients). Several patients did not receive antifungal treatment for candidemia (9.5%, 32/335), and in all cases, the patients died before or on the same day that the positive blood culture result was confirmed for *Candida* spp.

There were 352 *Candida* isolates identified, and *C. albicans* was the predominant species causing BSI (43.7%, 154/352). The second most prevalent species was *C. tropicalis* (21.3%, 75/352), followed by *C. parapsilosis* sensu lato (16.5%, 58/352), *C. glabrata* complex (8.5%, 30/352), and *C. krusei* (4.0%, 14/352). Other species included *C. lusitaniae* (n = 2), *C. famata* (n = 2), *C. guilliermondii* (n = 5), *Candida* spp. (n = 14), and *C. utilis* (n = 1), totaling 6.53% (Figure 1). Seventeen (5.1%) patients were infected by more than one *Candida* species. 

**FIGURE 1:**
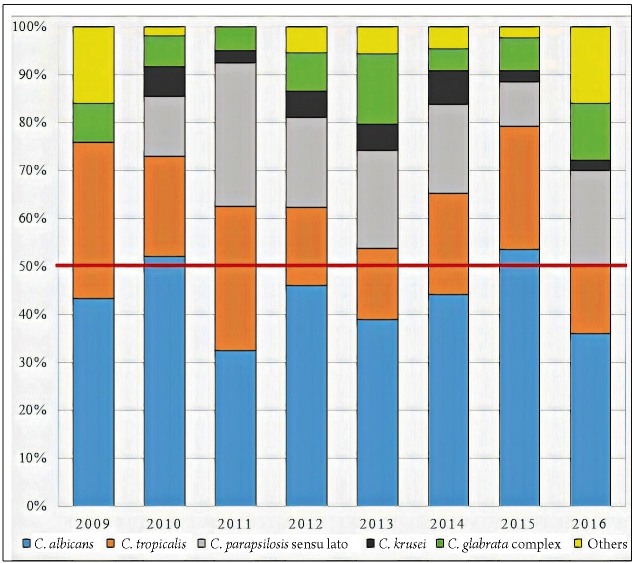
Distribution of the 352 identified isolates of *Candida* spp. over the 8 years of the study. The red line demonstrates the prevalence of non-*C. albicans* species. *C. lusitaniae, *C. famata*, *C. guilliermondii*, *Candida* spp., and *C. utilis*.

During the study, patients were at higher risk for mortality if they were hospitalized in the internal medicine unit, were elderly (>60 years old), were on hemodialysis, or required mechanical ventilation or orotracheal intubation. It has been previously reported that older patients are more likely to develop hospital infections because of the physiological changes associated with aging, a decline in immune response, and the need for invasive procedures^6^. When patients are subjected to procedures such as mechanical ventilation, orotracheal intubation, and hemodialysis, their microbiota becomes unbalanced and protective barriers are broken, thereby increasing the chance of colonization and nosocomial infection^7^.

The internal medicine unit is an infirmary where patients with difficult clinical conditions are treated, such as patients who have undergone coronary catheterization and patients who have acute and rare chronic diseases. These conditions depress the immune system and prolong hospitalization time, which increases the risk of infection. A multicenter study in Italy evaluated patients who were hospitalized for candidemia in the medical ward, concluding that patients with a mean age of 76 years with significant risk factors, such as immunosuppressive therapy, previous antibiotic therapy, diabetes mellitus, or severe sepsis, had a hospital mortality rate of 40.4%^8^. 

In this study, the use of parenteral nutrition was identified as a protective factor, representing a lower risk of mortality, thus demonstrating that nutritional care may reduce morbimortality rates caused by malnutrition as well as improve patient prognosis^9^. 

The use and duration of antifungal treatments (fluconazole and amphotericin B) were also protective factors. This emphasizes the efficacy of administration of appropriate therapy as a prophylactic or preemptive therapy or as soon as a diagnosis is confirmed^10^, considering all patients with confirmed candidemia who did not receive antifungal drugs died. Those patients who did not receive therapy as soon as the diagnosis was confirmed or in whom the diagnosis was delayed also died. Importantly, fluconazole is not routinely used as a prophylactic in the hospital under study. 

The total hospitalization time (days) and hospitalization in the emergency unit were also identified as protective factors. A shorter hospitalization time was directly proportional to a larger survival rate. In the emergency unit, the patient is quickly transferred to other specialized sectors, according to their clinical state.

Furthermore, patients with confirmed candidemia who were admitted to the pediatric ICU had lower mortality rates than did patients suffering from the same infection at other units of the hospital; the unit is a reference throughout the region because of rigid visitor control. The materials used for care are not shared (pressure device cuff, thermometer, stethoscope, among others), and sanitization of hand is a priority in patient care.

On the basis of other studies^1^ ^,^ ^11^, although *C. albicans* remains the most frequently encountered species in clinical laboratories, there has been an increase in the frequency of non-*C. albicans* species, such as *C. tropicalis*, *C. parapsilosis* sensu lato, *C. krusei*, and the *C. glabrata* complex. In this study, the *C. glabrata* complex increased over the years, whereas *C. albicans*, *C. tropicalis,* and (6)*C. parapsilosis* sensu lato remained constant. Non-*C. albicans* species are known for antifungal resistance, which reinforces the need to implement routine antifungal resistance testing at the study hospital, as it is not part of the current routine.

The incorporation of molecular methods for typing nosocomial pathogens has aided efforts to obtain a more fundamental evaluation of microorganisms. Establishing the clonality of pathogens can assist in source identification and distinguish between infectious and non-infectious strains^12^.

*C. parapsilosis* sensu lato presented significant results for death in this study. Over the last decade, the incidence of *C. parapsilosis* sensu lato has increased. The increased incidence has been attributed to a variety of risk factors, including the body’s selective growth capacity in hyperalimentation solutions and its high ability to colonize intravascular devices and prosthetic materials. In addition, patients who require prolonged use of central venous catheters or indwelling devices, such as cancer patients, are at increased risk for *C. parapsilosis* sensu lato infection^13^ ^,^ ^14^. 

The crude mortality rate was 54.6%, similar to that in several studies conducted in Brazil, China, and Pakistan (50.3-58%)^5^ ^,^ ^8^ ^,^ ^13^, and higher than that observed in other studies conducted in Brazil and China (37.0-38.1%)^15^ ^,^ ^16^.

The significant risk factors for mortality in patients who had candidemia were the requirement for invasive procedures (mechanical ventilation, hemodialysis, and orotracheal intubation), use of corticosteroids, and *C. parapsilosis* infection. Non-*C. albicans* species were the most prevalent causative agents of candidemia. In summary, the results of this study highlight the importance of total hospitalization time, the requirement for care related to invasive procedures, and actions to improve patient immunity, such as a good nutritional balance, which will contribute to reducing the severity of *Candida* infections and consequently, the morbimortality.
